# Bone Marrow Mesenchymal Stromal/Stem Cell-Derived Extracellular Vesicles Promote Corneal Wound Repair by Regulating Inflammation and Angiogenesis

**DOI:** 10.3390/cells11233892

**Published:** 2022-12-02

**Authors:** Gabriele Saccu, Valeria Menchise, Chiara Gai, Marina Bertolin, Stefano Ferrari, Cristina Giordano, Marta Manco, Walter Dastrù, Emanuela Tolosano, Benedetta Bussolati, Enzo Calautti, Giovanni Camussi, Fiorella Altruda, Sharmila Fagoonee

**Affiliations:** 1 Molecular Biotechnology Center “Guido Tarone”, Department of Molecular Biotechnology and Health Sciences, University of Turin, 10126 Turin, Italy; 2 Institute of Biostructure and Bioimaging, National Research Council (CNR), Molecular Biotechnology Center “Guido Tarone”, 10126 Turin, Italy; 3 Department of Medical Sciences, University of Turin, 10126 Turin, Italy; 4 Veneto Eye Bank Foundation (FBOV), 30174 Venice, Italy; 5 Ophthalmology Veterinary Practice, C.so Galileo Ferraris 121, 10126 Turin, Italy

**Keywords:** extracellular vesicles, cornea, MRI, Gd-AAZTA-MADEC, methylcellulose, mesenchymal stromal/stem cells, Esrp1

## Abstract

Severe corneal damage leads to complete vision loss, thereby affecting life quality and impinging heavily on the healthcare system. Current clinical approaches to manage corneal wounds suffer from severe drawbacks, thus requiring the development of alternative strategies. Of late, mesenchymal stromal/stem cell (MSC)-derived extracellular vesicles (EVs) have become a promising tool in the ophthalmic field. In the present study, we topically delivered bone-marrow-derived MSC-EVs (BMSC-EVs), embedded in methylcellulose, in a murine model of alkali-burn-induced corneal damage in order to evaluate their role in corneal repair through histological and molecular analyses, with the support of magnetic resonance imaging. Our data show that BMSC-EVs, used for the first time in this specific formulation on the damaged cornea, modulate cell death, inflammation and angiogenetic programs in the injured tissue, thus leading to a faster recovery of corneal damage. These results were confirmed on cadaveric donor-derived human corneal epithelial cells in vitro. Thus, BMSC-EVs modulate corneal repair dynamics and are promising as a new cell-free approach for intervening on burn wounds, especially in the avascularized region of the eye.

## 1. Introduction

The adult corneal epithelium plays a crucial role in preserving the transparency of the cornea, an avascularized and immune-privileged structure. Due to its anatomic position, the cornea is constantly amenable to injuries, and thus requires an inherent ability to repair tissue damage. Corneal healing is a complex and highly coordinated process that involves multi-layered regulatory scenarios to regenerate the damaged tissue and to achieve functional recovery [1]. Epithelial cell migration, proliferation and differentiation occur during this process, which also involves interaction with resident cells and factors secreted by the latter. Endogenous stem cell pools, such as limbal epithelial stem cells (LESCs), limbal mesenchymal stromal/stem cells (LMSCs) and the recently discovered corneal stromal stem cells (CSSCs), are engaged as a response to corneal injury, and all contribute to the repair and functional recovery of the corneal tissues [2].

Severe corneal damage can occur as a consequence of infections, systemic diseases, exposure to chemicals or environmental factors. All these factors, along with inefficient tissue-repair processes, trigger corneal neovascularization and scarring that can lead to a complete loss of vision, thereby compromising the patient’s quality of life and putting an immediate burden on the healthcare systems [3,4,5]. Current clinical approaches to manage corneal damage include use of lubricating fluids, antibiotics and autologous serum [6]. Prospective clinical studies have shown that in cases of severe damage, corneal transplantation is the gold standard, which assists in maintaining proper physiological function, thus avoiding blindness. Transplantation can be performed with tissue of autologous origin or with amniotic membrane [7]. However, as donated corneas are limited and vision loss is a devastating condition, there is an urgent need to develop alternative solutions. Moreover, if the patient’s limbal area is severely compromised by damage, corneal transplantation is not curative in the long term, as the regeneration and turnover of the corneal epithelium relies on limbal epithelial cells, which are absent in tissues derived from organ donors.

Tissue stem cells are important for the restoration of organ function. It was shown that several types of stem cells can participate in reinstating corneal homeostasis and transparency after corneal injury, including induced pluripotent stem cells and mesenchymal stem cells (MSCs) [3]. MSCs, due to their low immunogenicity and multilineage differential potential as well as immunomodulatory and paracrine functions, are optimal candidates for use in corneal wound-healing strategies [8]. Different delivery routes have been explored to maximize the number of cells reaching the injury site to achieve better therapeutic efficiency [9]. Topical administration offers the advantages of being non-invasive and patient-compliant due to the ease of application in long-term treatment. MSCs or their secretome administered to a rodent corneal epithelial wounding model has shown benefits in terms of attenuating corneal inflammation, reducing neovascularization, and promoting wound healing [10].

MSCs, however, can undergo lung entrapment and induce pulmonary vasculature embolism when injected systemically, react to chemokines present in the injured tissue microenvironment and may generate osteocytes and chondrocytes at the expense of target tissue-cell type, or elicit immune reactions despite their low immunogenicity [11,12,13]. Thus, the use of stem cells raises safety concerns in the clinical setting.

The MSC secretome, comprising a plethora of growth factors and cytokines, has also been shown to provide therapeutic benefits to rodents subjected to both mechanical and chemical corneal wounds [14]. Extracellular vesicles (EVs) are an integral part of the cell secretome. EVs are membrane-enclosed nano-sized particles that carry biomolecules such as RNA and proteins, which can be exchanged during intercellular and inter-organ communication. EVs play an important role in tissue regeneration after injury through reprogramming and the induction of pro-regenerative factors. Their biological function is expressed through proteins, lipids and nucleic acids, leading to a phenotypic response following ligand–receptor interaction, which can occur in three distinct ways: signaling, fusion and endocytosis [15,16]. In particular, MSC-derived EVs (MSC-EVs) have proven to be a valuable tool for various clinical applications in several areas, such as cardiovascular disease, acute kidney injury, liver disease, lung disease, skin wound healing and cancer suppression, involving several mechanisms [17,18,19,20,21]. The use of MSC-EVs in the ophthalmic field is also promising [22,23,24]. Recently, thrice-a-day topical application of human placenta-derived MSC-EVs on mouse corneas following alkali-burn injury showed pro-healing potential by modulating angiogenesis and stimulating corneal repair, and may offer a valid cell-free treatment alternative in ocular surface disease [1]. However, in order to improve patient compliance in the clinic, it is imperative to devise new drug delivery systems that can surmount the ocular surface barriers and release the therapeutic molecules for extended periods of time, thus enhancing regenerative efficacy and decreasing the frequency of applications.

In the present study, we topically delivered EVs in a murine model of alkali burn-induced corneal damage. The aim was to evaluate whether EVs derived from bone-marrow-derived MSCs (BMSC-EVs), applied in bio-adhesive and lubricant material that prolonged their effects in vivo and hence reduced the number of daily applications, could promote corneal repair. With the assistance of magnetic resonance imaging (MRI), an innovative approach to evaluate corneal angiogenesis in accordance with the 3R principles, we demonstrate that BMSC-EV treatment dampened corneal damage while controlling inflammation and neo-angiogenesis, which is essential for the repair of the avascularized cornea. Importantly, we also show that there are no side effects observed, despite the non-autologous nature of the EVs, due to the fact that the EVs are not internalized in the non-damaged corneas, indicating that as damage is resolved, these EVs no longer penetrate the corneal epithelium.

## 2. Materials and Methods

### 2.1. Cell Culture

Human corneal epithelial cells (HCECs) were obtained from the corneas of cadaveric donors, and were isolated and characterized as previously described [25]. Cells were maintained in CnT-Prime Epithelial Proliferation Medium (CellnTEC, Bern, Switzerland), 10% XerumFreeTM XF212 supplement medium (TNC Bio BV, Eindhoven, The Netherlands) and 1% penicillin-streptomycin (10 mg/mL, Life Technologies Corporation, Grand Island, NY, USA). Human BMSCs, purchased from Lonza (Basel, Switzerland), were cultured in mesenchymal stem cell basal medium (MSCBM, Lonza, Basel, Switzerland) as previously reported [26].

### 2.2. Isolation of EVs

BM-MSCs at 70% of confluence were washed and cultured in DMEM (Lonza, Basel, Switzerland) with penicillin/streptomycin and L-glutamine (Sigma-Aldrich, St. Louis, MO, USA) without FBS (Thermo Fisher Scientific Waltham, MA, USA) for 16 h at 37 °C and 5% CO_2_. Cell culture supernatants were centrifuged at 4000 rcf for 10 min at 4 °C followed by microfiltration using a 0.22 µm vacuum filter unit (Millipore, Burlington, MA, USA) to eliminate cell debris and apoptotic bodies. The supernatant was then centrifuged twice at 100,000 rcf, for 2 h at 4 °C in a Beckman Coulter Optima L-90K ultracentrifuge with rotor 45 Ti in polycarbonate tubes (355655; Beckman Coulter, Indianapolis, IN, USA). Pellets were resuspended in PBS (Lonza, Basel, Switzerland), supplemented with 1% DMSO (Sigma-Aldrich, St. Louis, MO, USA) and stored at −80 °C for further studies. EVs isolated from three donors (biological replicates) were characterized and used for in vitro experiments. BM-MSC-derived EVs (MSC-EVs) obtained from different donors were then pooled for in vivo treatments.

### 2.3. Nanosight Tracking Analysis (NTA) of EVs

EVs were analyzed by NTA, using the NanoSight LM10 system (NanoSight, Salisbury, UK). For each sample, three videos of 30 s duration were recorded, and camera levels were set for all the acquisition at 15. Briefly, EVs were diluted 1:200 in 1 mL of filtered saline solution (Fresenius Kabi, Bad Homburg vor der Höhe, Germany). NTA post-acquisition settings were optimized and maintained at a constant level among all samples, and each video was analyzed to measure mean EV size and concentration.

### 2.4. Characterization of EVs

EVs were characterized by flow cytometry analysis using the following fluorescein isothiocyanate (FITC)- or phycoerythrin (PE)-conjugated antibodies: CD73, CD105, CD44 (Miltenyi Biotec, Bergisch Gladbach, Germany). Conjugated mouse non-immune isotypic immunoglobulin G (IgG) (Miltenyi Biotec, Bergisch Gladbach, Germany) was used as control. Briefly, 10 µL of EVs were labeled for 15 min at 4 °C with antibodies and immediately diluted 1:3 and acquired. For analysis using the human cytofluorimetric bead-based MACSPlex exosome kit (Miltenyi Biotec, Bergisch Gladbach, Germany), the manufacturer’s protocol was followed [17]. Briefly, 1 × 10^9^ EVs were diluted in MACSPlex buffer (MPB) to a final volume of 120 µL, following which 15 µL of MACSPlex exosome capture beads (containing a cocktail of 39 different exosomal marker epitopes) were added to each sample. Samples were counterstained by adding 15 µL of a mix of APC-conjugated anti-CD9, anti-CD63 and anti-CD81 antibodies. After overnight incubation at 4 °C, beads were added for 15 min, samples were centrifuged and median fluorescence intensity (MFI) acquired. MFI was corrected, for all 39 exosomal markers, for medium background and gated according to their respective fluorescence intensity as per manufacturer’s instructions. All cytofluorimetric analysis were performed using the CytoFLEX flow cytometer (Beckman Coulter, Indianapolis, IN, USA) equipped with the CytExpert software version 2.3.0.84. For both classical flow cytometry and MACSPlex exosome kit, each analysis included 6 biological replicates.

### 2.5. Transmission Electron Microscopy Analysis (TEM) of EVs

Freshly prepared EVs were placed on 200 mesh nickel formvar carbon-coated grids (Electron Microscopy Science, Hatfield, PA, USA) and left to adhere for 20 min, followed by incubation with 2.5% glutaraldehyde containing 2% sucrose. Samples were negatively stained with Nano-W and NanoVan (Nanoprobes, Yaphank, NY, USA) and analyzed using a Jeol JEM 1010 TEM (Jeol, Tokyo, Japan).

### 2.6. Single Particle Interferometric Reflectance Imaging Sensor with ExoView^®^

MSC-EVs, at a final concentration of 1 × 10^8^, were pipetted onto a silicon custom chip coated with individual antibody spots against human CD9, CD63 and CD81. After overnight incubation, chips were incubated with a cocktail of fluorescent antibodies, anti-CD81 (green), anti-CD63 (blue) and anti-CD9 (red) and processed according to the manufacturer’s instructions (NanoView Biosciences, Brighton, MA, USA). Image and data acquisition for each chip were performed with the ExoView^®^ R100 (NanoView Biosciences, Brighton, MA, USA) machine and nScan acquisition software (NanoView Biosciences, Brighton, MA, USA). Data analysis was performed with NanoViewer 2.9.3 (NanoView Biosciences, Brighton, MA, USA) [27]. Analysis was performed on three spots for each capturing antibody in a single chip (four replicates).

### 2.7. Protein Measurement and Western Blot Analysis

MSC-EVs and cells were homogenized in RIPA buffer supplemented with a cocktail of protease and phosphatase inhibitors and phenylmethylsulphonyl fluoride (PMSF, Millipore Sigma, St. Louis, MO, USA). Protein concentration was measured by nicinchoninic acid (BCA) protein assay following the manufacturer’s instructions (Pierce, Thermo Fisher Scientific, Waltham, MA, USA). Five µg of protein extracts from EV or BM-MSCs were loaded onto 4–20% Criterion TGX Stain-Free Precast gels and transferred onto nitrocellulose membranes (BioRad, Hercules, CA, USA). The membranes were then incubated with the following primary antibodies: anti-CD9 (1:1000, Invitrogen, Carlsbad, CA, USA), anti-Alix (1:200; Santa Cruz, Santa Cruz, CA, USA), anti-RS29 (1:1000, Abcam, Cambridge, UK), anti TSG-101 (1:200; Santa Cruz, Santa Cruz CA, USA), anti-calnexin (1:1000, Abcam, Cambridge, UK), and the HRP-conjugated secondary antibodies: goat anti-rabbit (1:5000, Pierce, Thermo Fisher Scientific, Waltham, MA, USA), goat anti-mouse (1:5000, Pierce, Thermo Fisher Scientific, Waltham, MA, USA). Membranes were developed with SuperSignalWest Femto Maximum Sensitivity Substrate (Thermo Fisher Scientific, Meridian Rd., Rockford, IL, USA), and signal captured on a ChemiDoc™ MP Imaging System (BioRad, Hercules, CA, USA).

### 2.8. Animal Model and EV Therapy

An one-eye corneal alkali-burn-injury mouse model was generated as previously described [28]. FVB mice (3-month-old females bred in-house in specific pathogen-free conditions) were anesthetized with a mixture of 80 mg/Kg tiletamine and zolazepam and 16 mg/Kg xilazine, followed by the application of a drop of 0.5% proparacaine hydrochloride to the corneal surface for local analgesia. A paper disc (2 mm) soaked with sodium hydroxide (NaOH, 1N) was applied to the corneal surface of the right eye for 30 s, followed by extensive washing with PBS. Mice corneas were analyzed till day 14 following injury, histologically or by MRI, to assess tissue repair dynamics in our hands.

Ten µL of EV (10^9^ particles) resuspended in 2% PEG (P7181, Sigma-Aldrich, St. Louis, MO, USA) or carboxymethylcellulose (high viscosity 10 mg/mL, Sigma-Aldrich, St. Louis, MO, USA) was applied 15 min after injury, and on a twice-per-day regimen for up to 5 days following corneal injury. Thereafter, the EV-methylcellulose mixture was applied every other day, until day 14 (days 7, 9, 11 and 13, respectively). At least 3 mice were analyzed for each timepoint and experiment, as described below. This study protocol was approved by the Italian Ministry of Health (authorization number: 431/2017-PR and 372/2019-PR).

### 2.9. Histological Analysis

Mice were sacrificed and the injured and control corneas were collected. Formalin-fixed, paraffin-embedded corneal sections were stained with hematoxylin and eosin (H/E) or processed for IHC with anti-PCNA (1:300) antibodies (Santa Cruz Biotechnology, Dallas, TX, USA) as previously described [29]. For apoptosis analysis, an in situ cell-death detection kit, TMR-red (Roche Diagnostic GmbH, Mannheim, Germany) was used, according to the manufacturer’s protocol, and the slides were analyzed with a fluorescent microscope (VICO, Nikon Eclipse 80i). Cell nuclei were counterstained with DAPI.

### 2.10. Corneal Fluorescein Staining

A sodium fluorescein strip (Opitech, Prayagraj, U.P., India) was soaked in 1 mL of PBS and applied to the injured corneas to evaluate the epithelial repair process on days 0, 1 and 3. The extent of corneal damage was analyzed under UV light and photographed using a Moticam BTX8 camera (Motic, Xiamen, China) mounted on a Motic DM 143 stereo microscope (Motic Microscopy, Xiamen, China) at 4× magnification.

### 2.11. In Vivo EV Bio-Distribution

PKH26-labelled MSC-EVs were applied onto injured or uninjured control corneas for 4 h. After fixation in 4% paraformaldehyde (PFA), the cornea was dissected under a stereomicroscope, followed by further fixing in PFA. Corneal whole mounts were then stained with DAPI and phalloidin-FITC followed by analysis on a SP5 confocal microscope (Leica, Wetzlar, Germany). Images were acquired and reconstructed with Navigator Leica LAS-X software.

### 2.12. MRI Analysis

Subsequently, 7 T MRI scans were acquired on a Bruker Avance 300 spectrometer. T2W images were acquired using a standard Fast Spin Echo (FSE) sequence (rare factor = 8) with the following parameters (TR = 2500 ms, TE = 30 ms, NA = 8, FOV = 25 × 25 mm, slice thickness = 0.7 mm, matrix size 256 × 256). Subsequently, 1 T images were acquired on a 1 Tesla (T) MRI scanner (Aspect M2 High Performance System, Aspect Magnet Technologies Ltd., Letanya, Israel), consisting of a NdFeB Magnet equipped with a solenoid Tx/Tr coil with an inner diameter of 35 mm. T2W images were acquired using a FSE sequence, setting the following parameters: RT = 2500 ms, TE = 49 ms, matrix size = 160 × 160, slice thickness 1 mm, NA = 4, FOV = 40 mm. T1W images were acquired using gradient echo (GRE) sequence, setting the following parameters: RT = 40 ms, TE = 2 ms, matrix size = 128 × 128, slice thickness 1 mm, NA = 4, FOV = 40 mm, flip angle 60°. Since Gd-AAZTA-MADEC binds to albumin and is transported through the blood circulation, it can be used to study angiogenesis in injured corneas. A contrast agent (Gd-AAZTA-MADEC, Figure S3) was intravenously administered into a set of mice (n = 3). The mean signal intensity (SI) was determined on T1W images by drawing regions of interest (ROIs) on whole-eyeball, crystalline lens and corneal regions (using the XOR function, Figure S2) of mice with ImageJ software (http://rsb.info.nih.gov/ij/, accessed on 12 September 2022). Each SI value was normalized with respect to the noise signal and with the SI value of the same region in the image acquired before contrast-agent injection. The signal enhancement (SE%) between healthy and injured eye was monitored for 1 h after injection, using the following formula:

$$\mathrm{SE}\%=\frac{{\mathrm{SI}}_{\mathrm{injured}\;\mathrm{eye}}-{\mathrm{SI}}_{\mathrm{healthy}\;\mathrm{eye}}}{{\mathrm{SI}}_{\mathrm{healthy}\;\mathrm{eye}}}\times 100\;$$

The contrast agent (Gd-AAZTA-MADEC, Figure S3A) was thus intravenously administered into a set of mice treated with EV or with VHL (n = 6 for days 1 and 3, n = 5 for day 7) and SE% refers to SIs in images acquired 30 min after IV injection.

The thickness enhancement in injured eyes after alkali burn was calculated by measuring the corneal thickness on T2W images, using the following formula:

$$\mathrm{Thickness}\;\mathrm{enhancement}\;\left(\%\right)=\frac{{\mathrm{Thickness}\;}_{\mathrm{injured}\;\mathrm{eye}\left(\mathrm{mm}\right)}-{\mathrm{Thickness}}_{\mathrm{healthy}\;\mathrm{eye}\left(\mathrm{mm}\right)}}{{\mathrm{Thickness}}_{\mathrm{healthy}\;\mathrm{eye}\left(\mathrm{mm}\right)}}\times 100$$

### 2.13. RNA Extraction and qRT-PCR

Corneas were taken and snap-frozen for RNA extraction and gene-expression analysis. RNA was extracted using the PureLink RNA kit (Thermo Fisher Scientific, San Jose, CA, USA) and cDNA was prepared using a High-capacity cDNA Reverse Transcription kit (Thermo Fisher Scientific, San Jose, CA, USA). QRT-PCR was performed on QuantStudio 6 Flex (Thermo Fisher Scientific, San Jose, CA, USA). Inflammation and angiogenesis target gene expression was performed by SYBR Green^®^ Master Mix (Thermo Fisher Scientific, San Jose, CA, USA) and normalized to Gapdh expression. The primer sequences used in this study are listed in Table S1. A custom Esrp1 primer-probe was purchased and TaqMan™ qRT-PCR was performed and normalized to 18S expression (Thermo Fisher Scientific, San Jose, CA, USA).

### 2.14. In Vitro Scratch Wound Healing Assay

HCECs were seeded at 7 × 10^4^ cells/well in a 24 multi-well plate. At appropriate confluence, scratch wounds were generated and the cells washed thrice with PBS, and a complete medium containing MSC-EVs, at a concentration of 5000 EVs/cell, was added to the cells. Images were taken at different timepoints (every 6 h, up to 30 h) on a Zeiss time-lapse microscope (Carl Zeiss AG, Germany) at 10× magnification. The migratory effect was quantified by measuring the residual fractional wound area using ImageJ software (NIH, http://imagej.nih.gov/ij, accessed on 12 September 2022).

### 2.15. In Vitro TNFα Stimulation

HCECs were seeded at a density of 7 × 10^4^ cells/well in 24 multi-well dishes and stimulated after 4 h with 10 nM TNFα (Peprotech, Cranbury, NJ, USA). Following this, the complete medium, containing 5000 BMSC-EVs/cell, was added to the cells. After 24 h, RNA was extracted and gene-expression analysis was performed as described above.

### 2.16. Statistical Analysis

GraphPad Prism 8 software (San Diego, CA, USA) was used for the statistical analyses. Data are expressed as the mean and standard deviation (SD) in accordance with the data distribution. Parametric and non-parametric variables among the two groups were compared using Student’s unpaired *t*-test. Continuous and non-continuous variables among >2 groups were compared using analysis of variance (ANOVA). A *p*-value < 0.05 was considered statistically significant.

## 3. Results

### 3.1. Characterization of EVs Derived from Bone Marrow MSCs

**Figure 1:**
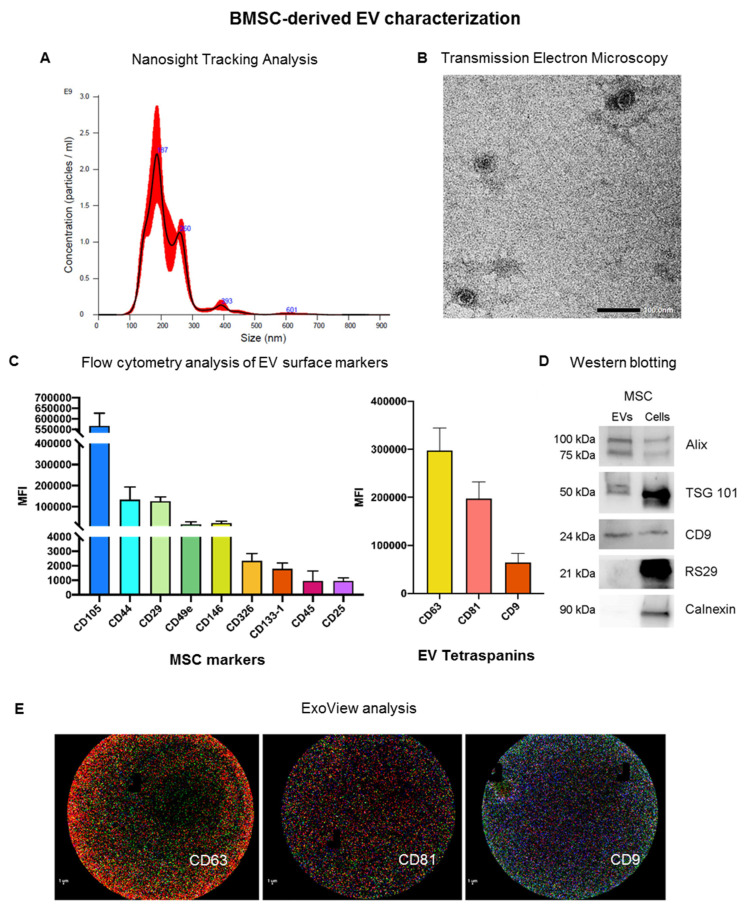
Characterization of BMSC-EVs. (**A**) Representative nanoparticle tracking analysis (NTA) showing EV size distribution, a pool of 3 samples being analyzed; (**B**) representative transmission electron microscopy image of MSC-EVs (scale bar = 100 nm) (n = 3, with each containing 3 technical replicates); (**C**) MACS multiplex bead-based flow cytometry analysis of different surface markers. MSC markers and EV tetraspanins are shown as mean fluorescent intensity (MFI) (n = 3, with each containing 3 technical replicates); (**D**) representative Western blot analysis comparing the expression in EVs (left column) and cells (right column) of EV markers, Alix, TSG101 and CD9, of intracellular markers, RS29, and calnexin as negative control for exosomes (n = 3, with each containing 3 technical replicates); (**E**) representative ExoView analysis of MSC-EVs showing, from left to right, anti-CD63 (red), CD81 (green), and CD9 (blue) capturing spot (scale bar: 1 µm), a pool of 3 samples was analyzed.

EVs were prepared from BMSCs, which were isolated and cultured as previously described, and starved overnight for EV collection by ultracentrifugation [26]. Pelleted EVs were then characterized according to the criteria suggested by the ISEV position paper [18] (Figure 1). The size distribution profile obtained by Nanosight tracking analysis demonstrated that BMSC-derived EVs (BMSC-EVs) have a mean size of 216.0 ± 3.8 nm with a yield of 2.26 e + 011 ± 2.15 e + 010 EV/mL (Figure 1A). Transmission electron microscopy analysis revealed membrane-enclosed vesicles with round morphology typical of EVs (Figure 1B). BMSC-EVs were also characterized using the MACS multiplex bead-based flow cytometry assay for the expression of surface proteins (Figure 1C). BMSC-EVs expressed the MSC markers CD105, CD44, CD29, CD49e and CD146, and the exosomal markers CD9, CD63 and CD81. The mesenchymal origin was further confirmed by the absence of expression of the endothelial marker CD31 (data not shown). The presence of EV markers (Alix, TSG101 and CD9) was confirmed at the protein level (protein content of 26.3 ± 8.1 g/10^9^ EVs for BMSC-EVs) by Western blotting analysis (Figure 1D). EVs were negative for RS29 and calnexin, confirming the absence of ribosomal and cytoplasmic components in isolated EVs. Furthermore, the recently developed Exoview technology was used to characterize BMSC-EVs, and confirmed the presence of the EV markers CD63, CD81 and CD9 (Figure 1E). Overall, these results show that EVs were enriched from BMSC culture supernatant.

### 3.2. MRI-Based Corneal Wound Repair Dynamics in Alkali-Burn Mouse Model

A one-eye alkali-burn model of corneal injury was induced in FVB mice by applying a sodium-hydroxide-solution-soaked paper disc on the right eye for 30 s followed by copious rinsing, as previously described [28]. The injured and control corneas were monitored in vivo by MRI up to day 14, and compared with histological data upon sacrifice (data not shown). MRI T2 weighted (T2W) images revealed a slight edema in the right eye from the day after the alkali burn, resolving on day 14 (Figure S1).

On day 14, the injured right eye had compromised corneal transparency (Figure S1, inset) compared to untreated controls. We thus proceeded with investigating the potential therapeutic effects of BMSC-EVs on corneal wound repair.

### 3.3. Enhanced Corneal Wound Repair Following BMSC-EV Treatment In Vivo

**Figure 2:**
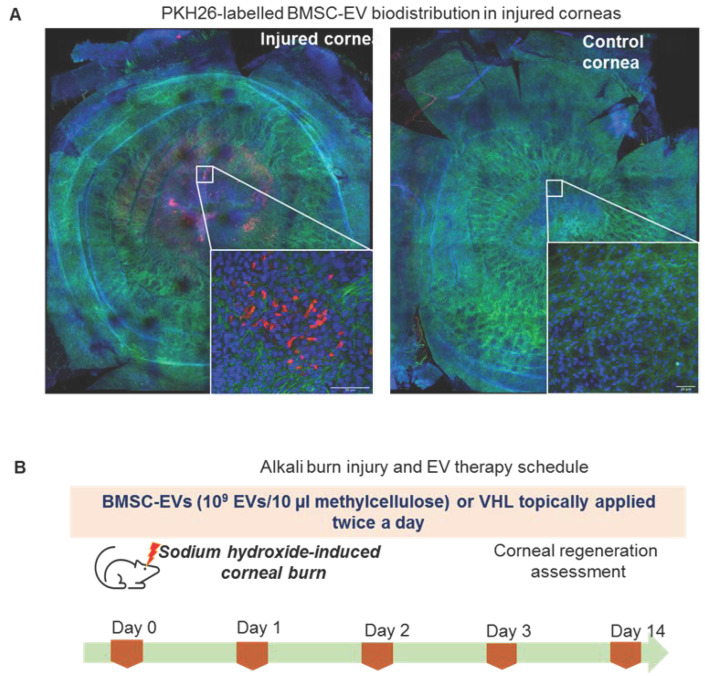
MSC-EV treatment of murine corneas upon alkali burn. (**A**) Confocal microscopy images of corneal whole mounts, reconstructed using the mosaic function of Leica Sp5 confocal microscope, following treatment of the injured or uninjured control corneas with PKH26-labelled (Red) MSC-EVs at 10× magnification. Inserts are shown at 40× magnification (scale bar: 50 nm). F-actin and nuclei were stained with phalloidin-FITC (green) and DAPI (blue), respectively. Representative images are shown (n = 2, biological replicates). (**B**) Alkali burn induction and EV treatment schedule in mice. Corneal regeneration was assessed during a 14-day time course.

BMSC-EVs were topically applied 15 min after the alkali burn to reproduce the slight delay in treating corneal burns that occurs in real life following chemical-based hazards. BMSC-EVs, at a dose of 1 × 10^9^ EV/10 µL of PEG or carboxymethylcellulose (methylcellulose), were used. EVs delivered in PEG did not show any significant therapeutic effect on wound repair in alkali-burned corneas with respect to PEG alone (data not shown). Methylcellulose, which is also employed in ophthalmic solutions, was therefore employed as a vehicle (VHL) for EVs [30]. The bio-distribution of PKH26-labelled BMSC-EVs, applied in methylcellulose, 15 min after the alkali burn, was analyzed. The data show that the labelled EVs were present in the damaged area 6 h after topical application (Figure 2A). EVs were internalized into the first corneal epithelial layers as shown in the cross section of the confocal image (Figure S2).

**Figure 3:**
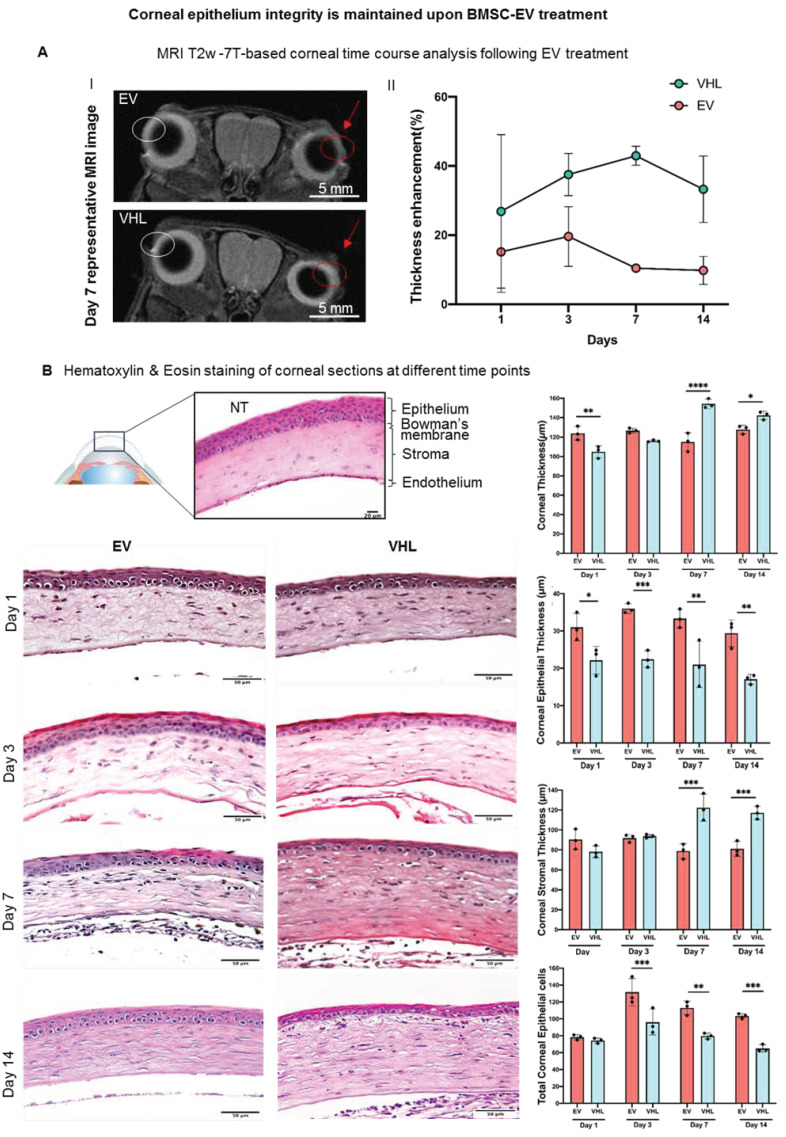
MRI-based and histological analyses of injured corneas. (**A**) MRI T2w-based time course analysis of corneal thickness following treatment with BMSC-EVs and VHL. Representative images obtained at day 7 post BMSC-EV treatment versus VHL control is shown. The damaged eye is highlighted by a red arrow and red circle. The uninjured contralateral eye is indicated by a white circle. The graph shows quantification of corneal injury thickness at different timepoints after treatment with BMSC-EVs and VHL; n = 2. (**B**) H/E staining of corneal sections showing a representative time course at days 1, 3, 7 and 14 following BMSC-EV or VHL treatment of injured cornea, compared to a normal one (NT). Scale bar = 50 µm. The graphs show difference in thickness of the different corneal layers and the total number of epithelial corneal cells (n = 3 representative of 2 independent experiments). Two-way ANOVA analysis was performed to evaluate statistical significance (* *p* < 0.05, ** *p* < 0.01, *** *p* < 0.001, **** *p* < 0.0001). ● represents biological replicates.

BMSC-EVs or VHL were then topically delivered on a twice-daily basis till day 14 post alkali burn (Figure 2B). MRI analysis of the mice corneas over time revealed corneal thickening where the alkali-soaked paper discs were applied (Figure 3A). Instead, the thickness of epithelial compartment decreased, likely as a consequence of the inefficient tissue repair process, as indicated by histological analyses. In fact, at day 1, the BMSC-EV-treated corneas had a multi-layered epithelium compared to VHL-treated ones, which had visibly reduced corneal epithelium thickness (Figure 3B).

**Figure 4:**
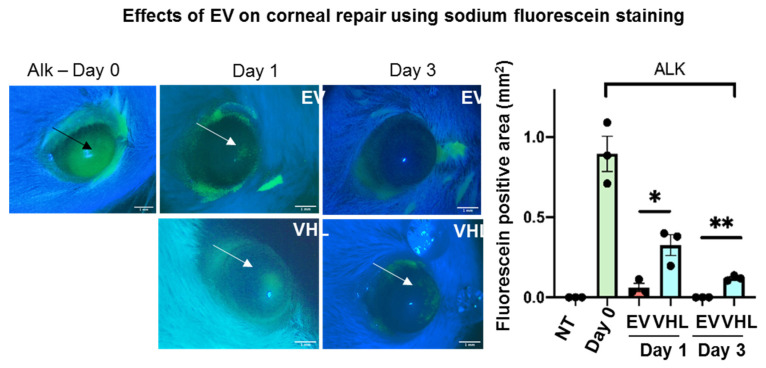
Effects of MSC-EVs on corneal wound repair. Fluorescein staining in vivo immediately following damage induction (ALK day 0; the black arrow shows NaOH-induced damaged area). Damage resolution till day 3 is shown (arrows indicate positivity for fluorescein). The graphical representation of wound repair at days 0, 1 and 3 following alkali burn is shown. Fluorescein-positive areas (mm^2^) were measured and mean ± SD is shown; n = 3 (representative of 2 independent experiments), * *p* < 0.05, ** *p* < 0.01. Scale bar = 1 mm. ● represents biological replicates.

This difference in epithelial thickness was maintained until day 14 post treatment. Since the repair of the corneal epithelium in this model was observed around day 3, as indicated by sodium fluorescein stain (Figure 4), we subsequently delivered BMSC-EVs or VHL topically, on a twice-daily schedule, until day 3 post alkali burn.

**Figure 5:**
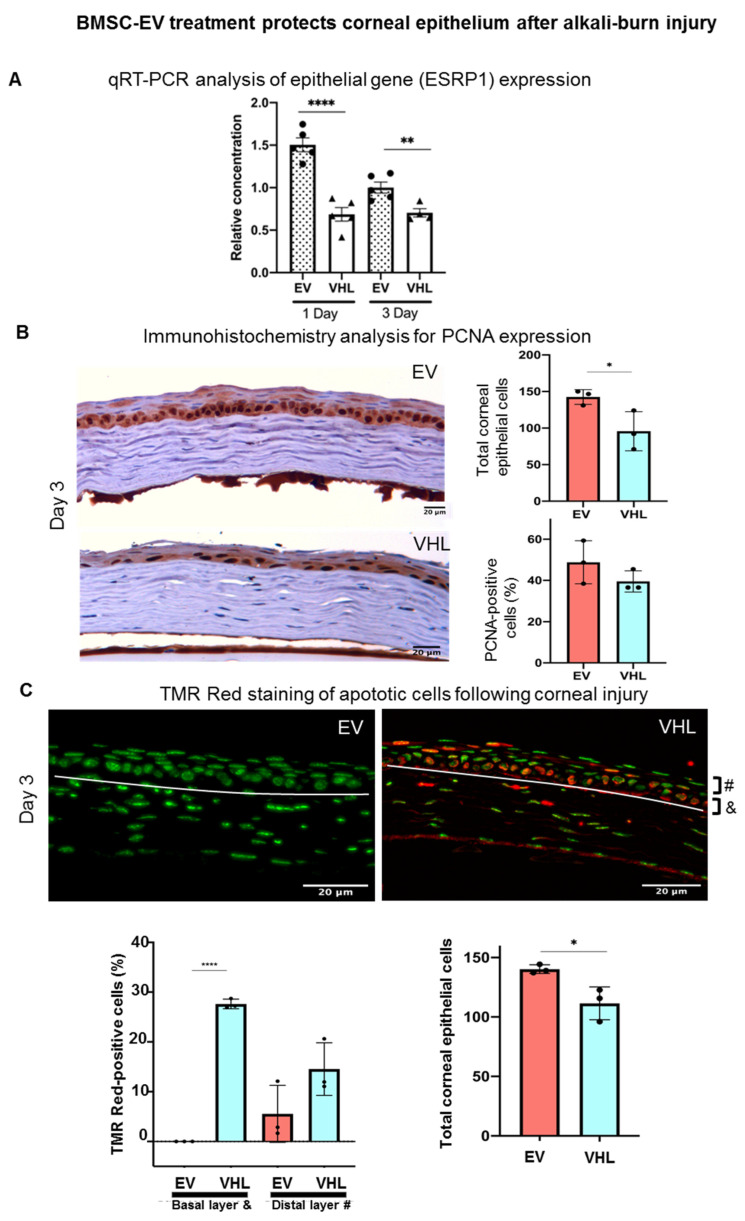
BMSC-EV treatment protects the corneal epithelium after alkali-burn injury. (**A**) The expression of Esrp1, a marker of epitheliality, is shown at day 1 and day 3 post-EV or VHL treatment in the injured corneas; n = 5, ** *p* < 0.01, **** *p* < 0.0001. ● represents biological replicates with EVs treatment; ▲ represents biological replicates with VHL treatment. (**B**) Immunohistochemistry analysis of PCNA expression in corneal epithelium at day 3 after treatment with EV or VHL (magnification 20×). Data are expressed as mean ± SD; n = 3, * *p* < 0.05, Scale bar = 20 µm. (**C**) Representative TMR Red-stained corneal sections at day 3 post-alkali burn are shown (magnification 20×; scale bar: 20 µm). The white line demarcates the stroma from the epithelium. Apoptotic cells (red) and nuclei (green) are shown in BMSC-EV- and VHL-treated corneas. Scale bar = 20 µm. The white line represents the epithelium-stroma borderline. Graphs show quantification of apoptotic cells in the basal (&) and more distal (#) layers of the cornea. Data show mean ± SD; n = 3, * *p* < 0.05, **** *p* < 0.0001; and quantification of total epithelial cells (n = 3, * *p* < 0.01). ● represents biological replicates.

In particular, at day 3, the BMSC-EV-treated group showed a significant reduction in fluorescein-positivity, suggesting a faster recovery of epithelial integrity relative to VHL controls. Moreover, quantitative real-time PCR (qRT-PCR) analysis of the mRNA expression levels in whole corneal tissues of epithelial splicing regulatory protein 1 (*Esrp1*), an epithelial-specific gene, indicated a higher abundance of the epithelial compartment in EV-treated samples (Figure 5A). In order to assess whether BMSC-EVs influenced the proliferation of corneal cells, PCNA staining was performed (Figure 5B). No statistically significant differences in PCNA-positivity were observed. However, the number of total corneal epithelial cells was significantly higher in BMSC-EV- vs. VHL-treated corneas. Importantly, in the basal layer responsible for maintaining the proliferative epithelial cell pool in the cornea, TMR-red positive (dead) cells were absent in injured corneas treated with EVs compared to controls, and the difference was statistically significant at day 3 (Figure 5C). These results indicate that BMSC-EV treatment counteracts epithelial cell loss following corneal injury by exerting, at least in part, a pro-survival activity.

**Figure 6:**
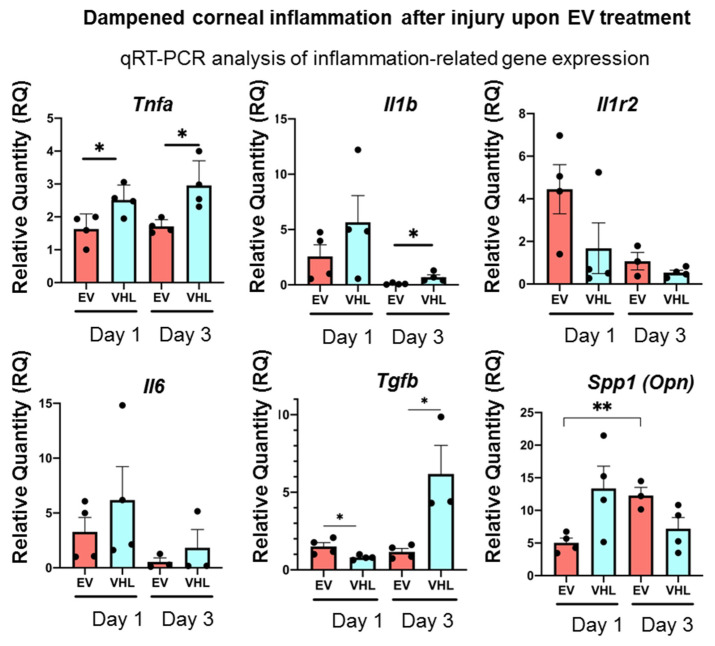
Dampened corneal inflammation after injury upon BMSC-EV treatment. Inflammation-related gene expression assessed by qRT-PCR at different timepoints post-alkali burn injury and upon BMSC-EV- and VHL-treatment. Mean ± SD is shown and is representative of 2 independent experiments (n = 4, * *p* < 0.05, ** *p* < 0.01). ● represents biological replicates.

As the MSC secretome, including EVs, is known to promote tissue regeneration through its immunomodulatory action, we further analyzed the inflammatory status of the alkali-burned corneas following BMSC-EV treatment [31,32]. As shown in Figure 6, the expression of the key pro-inflammatory cytokines, *Tnfα*, *Il-1β* and *Il-6*, were strongly dampened in the BMSC-EV-treated mice corneas as early as day 1 following alkali-induced injury compared to VHL-treated controls.

On the other hand, *Tgfβ*, a pleiotropic cytokine, showed constant expression in the BMSC-EV-treated corneas up to day 3 following alkali burn, unlike VHL-treated ones, in which *Tgfβ* expression increased statistically significantly at day 3. The expression of Il1-R2, a negative regulator of the IL-1 cascade, did not change significantly, while that of *Spp1* (Osteopontin, *Opn*), previously shown to be involved in corneal wound healing following alkali burns, showed a statistically significant increase in the corneas from day 1 to day 3 after EV treatment compared to VHL application. These data show that BMSC-EV treatment triggers an anti-inflammatory gene-expression program in injured corneas [33,34].

### 3.4. BMSC-EVs Counteract Neoangiogenesis in the Injured Cornea

**Figure 7:**
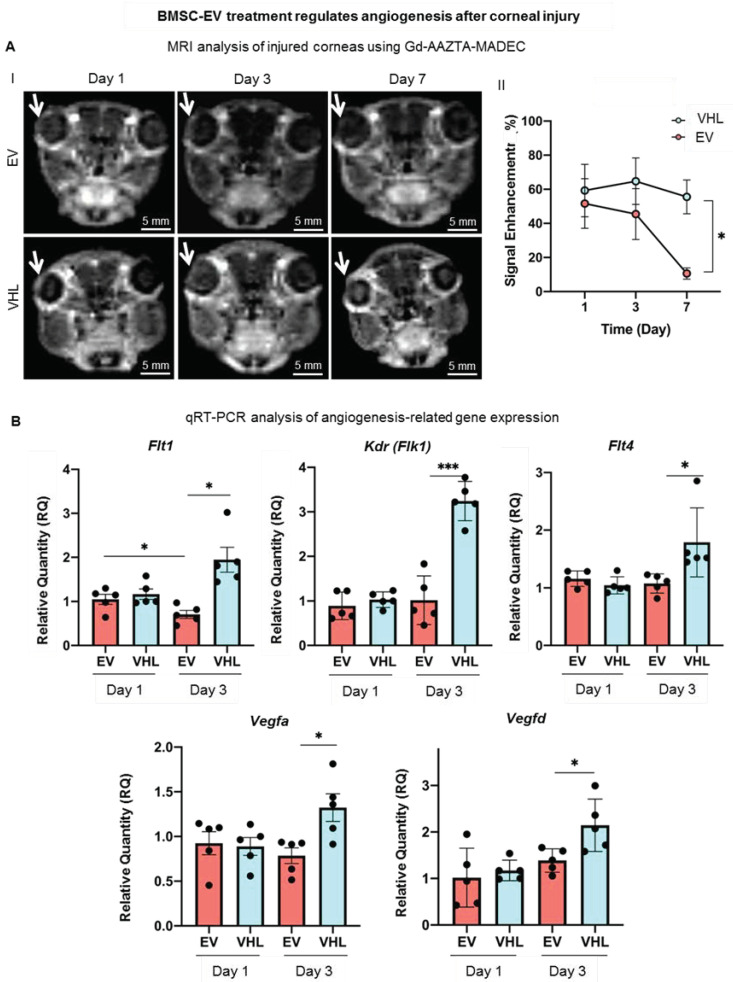
Neoangiogenesis assessment in corneas subjected to alkali burn. (**A**) Representative MRI T1-weighted images of Gd-AAZTA-MADEC-injected mice corneas revealing angiogenic process at days 1, 3 and 7 after BMSC-EV or VHL treatment. White arrow represents the injured area. Graph shows the measured corneal MRI signal enhancement (see Figure S3 for further details) (day 1: n = 6, day 3: n = 6, day 7 n = 5; mean ± SD, * *p* < 0.05). (**B**) Gene expression analysis of angiogenesis- and lymphangiogenesis-associated factors at days 1 and 3 after EV or VHL treatment in alkali-burned corneas. Data show mean ± SD; n = 5; * *p* < 0.05, *** *p* < 0.001. ● represents biological replicates.

In order to assess whether BMSC-EV treatment could regulate the neo-angiogenesis that occurs following corneal burn injury, we performed MRI with a blood-pool contrast agent (Gd-AAZTA-MADEC or MADEC) for 7 days to assess whether the contrast agent generates a significant signal increase in the corneal area (Figure 7A) [35].

We expected an increase in the signal intensity likely related to the neoangiogenetic process in the corneal district, as MADEC remains confined inside the vessels. An experimental set-up was performed (Figure S3). T1 weighted (T1W) MRI images taken at day 1 showed that the injection of MADEC (Figure S3A) elicited a remarkable immediate increase in signal intensity in the injured right eye compared to the healthy one (Figure S3B). The signal further increased for a short time (from 20–25 to 30 min following IV injection of MADEC) and finally decayed after 1 h, due to the clearance of the contrast agents from corneal blood circulation (Figure S2C). Thus, 30 min after the IV delivery of MADEC was chosen for the detection of the maximum MRI signal in the corneal district. The MRI analysis was then performed on two groups of mice, treated with BMSC-EVs or VHL, and angiogenesis was followed on the corneal surface at days 1, 3, 7 after alkali burn (Figure 7A). The MADEC-induced signal intensity enhancement in right eyes was found to be consistently reduced with respect to the contralateral non-injured ones in BMSC-EV-treated mice, as compared to the VHL control group, and it gradually decreased over time, becoming statistically significant at day 7 (Figure 7A).

As a higher expression of *Vegf* and *Flt1* has been reported in inflamed and vascularized corneas [18], angiogenesis-related gene expression was also evaluated. The expression of the growth factors *Vegfa* and *Vegfd* was found to be statistically significantly upregulated at day 3 in the VHL controls compared to EV-treated injured corneas (Figure 7B). The expression of the VEGF receptors, *Flt1*, *Kdr* (*Flk1*) and *Flt4* also followed a similar trend, increasing significantly only in the VHL-treated injured corneas at day 3 with respect to EV-treated ones. These data show that BMSC-EVs, delivered in methylcellulose, counteract the neo-angiogenetic process that occurs after alkali-burn injury in the cornea.

### 3.5. BMSC-EV Treatment Enhances Wound Closure of Human Corneal Epithelial Cells In Vitro

**Figure 8:**
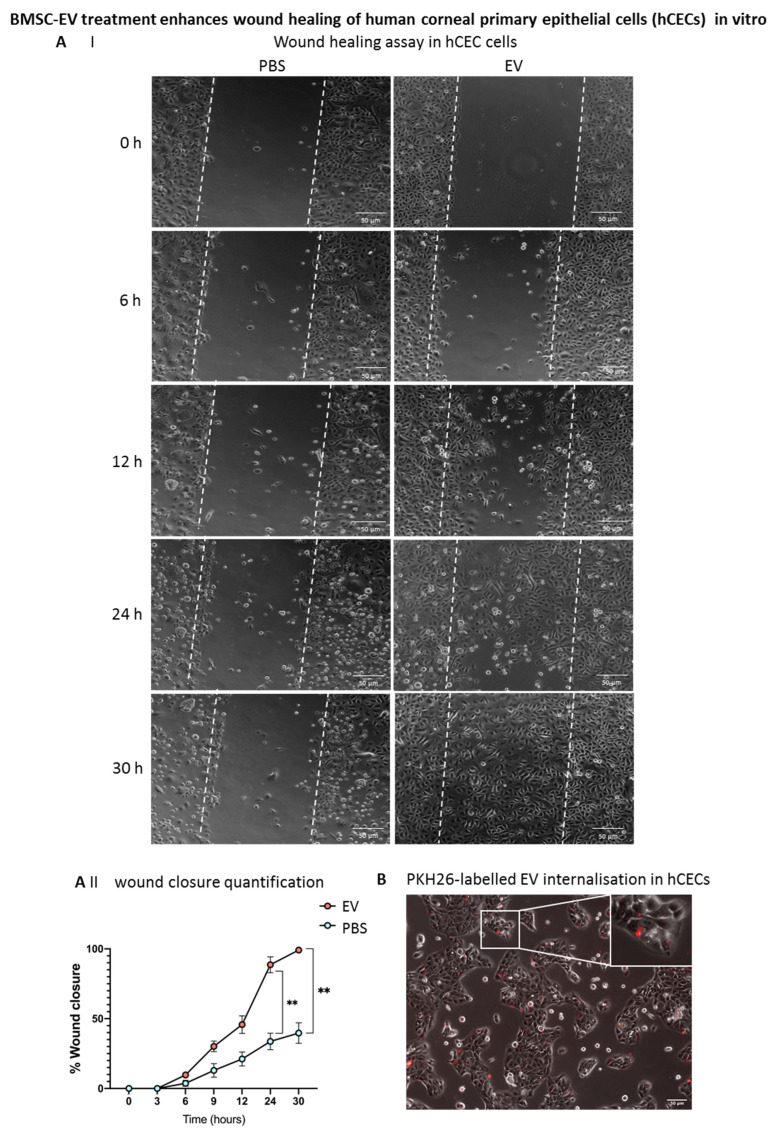

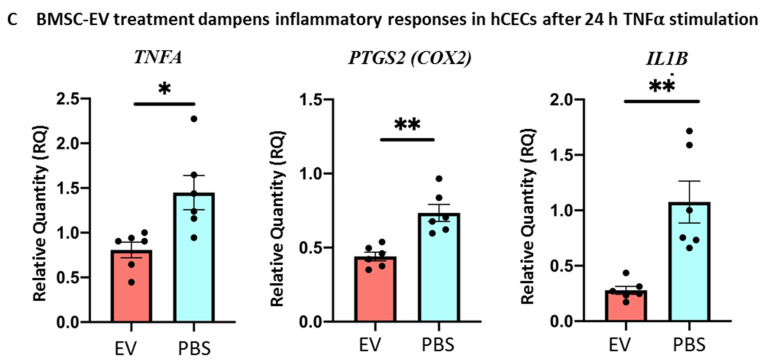
Effects of BMSC-EV treatment on primary human corneal epithelial cells (HCECs) in vitro. (**A**) Representative images of the wound-healing assay. Wound closure dynamics of HCECs after treatment with BMSC-EVs or PBS at different timepoints are shown. The white dashed lines are the edges of the cell migration at 0 h. Scale bar = 50 µm. The graph shows quantification of wound repair. Data represent mean ± SD; n = 5, ** *p* < 0.01. ● represents biological replicates (**B**) HCECs were treated with a single dose of 5000 PKH26-labelled EVs/cell. EV internalization in HCECs 24 h after treatment is shown (red signal). Insert shows larger magnification of selected area. Scale bar = 50 µm. (**C**) qRT-PCR analysis of inflammation-related gene expression in HCECs treated with TNFα for 4 h, followed by treatment with a single dose of EVs at 5000 EVs/cell or PBS for 24 h. Data show mean ± SD; n = 6; * *p* < 0.05, ** *p* < 0.01. ● represents biological replicates.

Human corneal epithelial cells (hCECs) were obtained from cadaveric donors not suitable for transplantation and characterized as previously described [25]. A scratch assay was performed on confluent hCECs, and the effect of BMSC-EVs on wound closure in vitro was assessed. Interestingly, BMSC-EVs induced a faster wound closure with respect to PBS-treated controls, as early as 6 h after the scratch formation, with a statistically significant difference at 24 and 30 h post-wounding (Figure 8A). In order to confirm that the difference observed in wound closure was due to BMSC-EV treatment, PKH26-labelled EVs were applied to hCECs. As shown in Figure 8B, positivity for PKH26 was found in hCECs after 4 h of treatment, suggesting that, in these cells, EVs can influence the dynamics of wound closure.

Furthermore, an inflammatory milieu quite similar to the in vivo situation after alkali burn was mimicked in hCECs in vitro by treating the cells with TNFα for 4 h. Importantly, treatment with BMSC-EVs dampened the TNFα-induced inflammatory response in hCECs compared to PBS controls (Figure 8C). In particular, the expression of *TNFA*, *COX2* and prostaglandin-endoperoxide synthase 2 (*PTGS2*) were significantly reduced following EV treatment versus controls, showing the capacity of BMSC-EVs to counteract an inflammatory response in these cells, hence supporting our in vivo data.

## 4. Discussion

Preclinical studies have amply demonstrated how stem cells and their secretome are therapeutically apt in the treatment of injuries by modulating proliferation, apoptosis, inflammation, angiogenesis and fibrosis in several organs such as the liver, kidney, skin and cancer, as well as in the ophthalmic field [9]. EVs especially have become an exciting, next generation, cell-free approach for alleviating both acute and chronic disturbances [36]. EVs from human MSCs, for example those derived from human pluripotent cells (hP-MSCs) or the human cornea, have been employed in preclinical studies as a prelude to their application in ophthalmological disorders [23]. Hitherto, only three clinical studies have employed MSC-EVs for macular holes, dry eye diseases and retinitis pigmentosa (source: https://clinicaltrials.gov/, accessed on 30 November 22; NCT03437759, NCT04213248 and NCT05413148, respectively). In the active and recruiting NCT04213248 study, umbilical MSC (UMSC)-derived exosomes will be applied at a dose of 10 µg/drop of UMSC exosomes four times a day for 14 days after two weeks’ application of artificial tears, in order to evaluate the progression of dry eye in patients with chronic graft-versus-host diseases [37]. To our knowledge, no clinical studies regarding the use of EVs or exosomes for eye injuries/burns have yet been undertaken. In the present work, we show the promise of BMSC-EVs, delivered in methylcellulose, for the treatment of chemically-induced corneal burn injury in mice. Our data demonstrate that BMSC-EVs, topically applied on a twice-daily basis early after a burn injury, counteract corneal epithelial cell death, neo-angiogenesis and inflammation programs, thus significantly limiting damage to the corneal surface and associated pressure on the ocular system.

EVs offer innovative therapeutic and diagnostic opportunities for unmet clinical needs, especially due to their size, biocompatibility, ability to cross physical and biological barriers, quasi-inexistent toxicity and immunogenicity and biomolecular cargo [38]. However, the bioavailability of EVs depends extensively on the delivery routes, dose and frequency of administration as well as clearance rates in vivo [9]. Several preclinical studies have reported on the rapid clearance of intravenously-injected EVs due to uptake by immune cells, so that delivery of a therapeutic dosage of EVs to the target organ is not possible [39]. Analogously, EVs topically administered on a skin or ocular surface are subjected to rapid clearance imposed by high sweat or tear turnover, respectively [40]. Thus, the use of an appropriate scaffold can reduce the number of daily topical applications of EVs and hence improve patient compliance. To avoid such a loss of EVs, polymers, such as synthetic poly (ethylene glycol) (PEG), have been used to tailor EVs to inhibit their interaction with blood components and to increase their circulation time and non-specific uptake [41]. In this study, we initially topically applied PEGylated BMSC-EVs on the injured corneas but could not observe any difference in wound-healing dynamics, probably due to the slower and more controlled release of PEG-stabilized EVs. In fact, it has been shown that drug release from PEG formulations can last up to 14 days, while that from cellulose-based biomaterials can last up to 12 h [42]. Thus, the bio-adhesive and lubricant methylcellulose, currently employed in ophthalmic eye drops against dry eye disease, was used for EV application in an attempt to enhance their biological effects in vivo [43]. Importantly, the methylcellulose-EV mixture successfully released EVs in the injured areas of the cornea, as evidenced by biodistribution studies on corneal whole mounts, probably because the endogenous moisture increased external adsorption with respect to PEG (which is used mostly in settings in which the slow release of biomolecules is required and reduces compression after drying), thus allowing better EV bioavailability [44,45]. The methylcellulose formulation is thus promising for developing an EV-based innovative ophthalmological intervention [46]. In this setting, EVs can be easily visualized in the cytosol, as shown by the colocalization with F-actin, suggesting a possible EV internalization through incorporation by the plasma membrane. Other formulations have shown promising results in preclinical models of corneal damage. For instance, Tang et al. used EVs prepared from induced pluripotent stem cell (iPSC)-derived MSCs resuspended in biocompatible and thermosensitive chitosan-based hydrogels to assess their therapeutic effects on corneal epithelium and stroma regeneration in rats with anterior lamellar damage [47]. They showed that this hydrogel promoted a sustained release of EVs, which enhanced the repair of the injured corneas.

EVs derived from MSCs of different origin bear wound-healing properties. IPSC-derived MSC EVs, for example, offer the possibility of preparing autologously derived EVs for the long-term treatment of ocular diseases, hence avoiding immunological reactions [47]. Tissue-derived MSCs, on the other hand, have been extensively studied in vivo, revealing how their secretome is promising in tissue regeneration. Several studies have shown that MSC-EVs can induce angiogenesis, the fine-tuning of which is critical for wound repair in several tissues [48]. A recent systematic review and meta-analysis, investigating the therapeutic efficacy of MSC-EVs in animal models of diabetic wounds, showed a significantly greater number and density of blood vessels in rodents treated with MSC-EVs with respect to controls [49]. However, using EVs that do not stimulate angiogenesis is essential for the wound repair of the avascular cornea. Compared to adipose tissue MSC-derived EVs (ADSC-EVs), which contain angiogenesis-promoting biomolecules important for the wound healing of tissues such as the skin, the BMSC-EVs had pronounced effects on re-epithelialisation, proliferation, cell adhesion and metabolic processes, most probably due to the absence of miRNAs involved in angiogenesis, such as HIF-1 signaling pathway-associated miRNAs as well as *miR-210* and *miR378* [26]. We thus employed EVs isolated from BMSCs, well characterized in our laboratory, to investigate their therapeutic effects on corneal wound repair [26]. Our MRI and gene-expression data show reduced neovascularization in injured murine corneas following treatment with BMSC-EVs compared to VHL controls. The TGFβ signaling cascade is an important modulator of EV pro-angiogenetic activity [50,51]. Interestingly, our model evinces that TGFβ signaling is not induced in the injured corneal tissue upon the topical application of BMSC-EVs. This is supported by the lack of induction of TGFβ-target genes, *Vegfa* and *Vegfd*, and *Kdr* (*Flk-1*), as well as *Flt-1* and *Flt-4* expression in the injured corneas following BMSC-EV treatment compared to controls [52]. Thus, the correct choice of EV source is important when considering regeneration in a tissue-dependent context, as angiogenesis promotion is not part of the corneal wound-repair process.

Upon superficial alkali burn, corneal epithelial cells are the most prominently affected and are first ones to undergo apoptosis and detach from the underlying Bowman’s membrane [53]. The rapid restoration of an intact epithelium in the injured area or prevention of epithelial cell loss is critical for corneal wound healing [54]. This is a highly regulated process involving the proliferation and migration of epithelial and stromal cells. Our data show that, in the alkali-induced-injury model, treatment with BMSC-EVs prevented epithelial cell loss by inhibiting cell death at 3 days post-injury. This was confirmed by the high expression of the epithelial gene, *Esrp1*, as well as the number of epithelial cells present in the EV-treated corneas compared to the VHL-treated controls. The epithelial layer maintenance in the EV-treated group was not dependent on cell proliferation. This is in line with previous findings, whereby EVs derived from BMSCs protected renal tubular epithelial cells from apoptosis in different mouse models of acute kidney injury through an RNA-based mechanism [55,56]. EVs derived from MSCs from different sources carry several important anti-apoptotic microRNAs (miRNAs) such as *miR-21/-30/-125b/-130a/ 199a/-210/-223/-242*, or proteins, such as PCNA, BCL-XL and BCL-2 [57,58]. Moreover, several studies have investigated and reported on the functional proteins inside EVs by MS/MS proteomic analysis, highlighting the presence of proteins involved in proliferation, cell adhesion and other key signaling pathways [59,60]. Work by Pomatto et al. has revealed that BMSC-EVs contain proteins involved in inflammation, cell adhesion, fibrosis and apoptosis, such as IL13, IL1RL2, IL5, IL10, FN1, ENPP2 and GH1. In addition, proteins of the TGFβ superfamily, GDF3 and GDF11, known to modulate apoptosis in cancer, were also found in the BMSC-EVs [26,61,62]. Other proteins, such as S1PR1, present in BMSC-EVs, are also involved in the inhibition of apoptosis via the STAT3 pathway [63]. Furthermore, BMSC-EV delivery in an inflamed colon model reduced the cleavage of caspase-3, -8 and -9, hence suppressing apoptosis in this setting [64]. All these data explain how the application of BMSC-EV on injured corneas could protect against the epithelial cell loss observed in our model.

Inflammation is one the consequences of chemical-burn-induced corneal damage [65]. The absence of an inflammatory state in the corneal microenvironment is essential for successful damage repair. If inflammation is not resolved immediately, it may lead, with time, to corneal vascularization and fibrosis. In our model, inflammatory markers were decreased in the corneal wounds receiving BMSC-EVs with respect to controls. The administration of BMSC-EVs allows us to instate an anti-inflammatory microenvironment through the delivery of anti-inflammatory proteins such as IL10 and IL5, IL1R2 [26], or the suppression of pro-inflammatory proteins by growth-related oncogene (GRO), as observed in a mouse model of dry eye disease [66,67]. In an inflammatory eye model, BMSC-EV-derived indoleamine 2–3 dioxygenase (IDO) promoted and established an immune-suppressive and anti-inflammatory environment mediated by T-regulatory cells producing TGFβ and IL10 [68]. In addition to their anti-inflammatory effect, BMSC-EVs promoted the repair and survival of injured neurons (neuritogenesis of retinal ganglion cells) [68,69].

Corneal homeostasis is a dynamic process that must be preserved or blindness will result. This study represents a novel cell-free perspective in the resolution of corneal injury through BMSC-EV administration. Importantly, our data show that BMSC-EVs orchestrate corneal homeostasis by modulating key processes such as cell survival, migration and inflammation, following injury, without adverse events. Our results are backed up by the work of Yu et al., who showed, on a human cornea-on-a-chip composed of human epithelial and endothelial corneal cells, that treatment with BMSC-EVs significantly reduced inflammation and neovascularization in an in vitro mild corneal scratch wound-healing model by controlling the expression of the host pro-inflammatory cytokine, matrix metalloprotease-2 [70]. It has been shown in a mouse model of severe skin burn injury that there is a biphasic release of EVs in the circulation following burn damage, with the EVs released early following burn injury promoting proinflammatory and tissue damaging responses, while those released at later stages after burn contribute to the impaired responses to infection [71]. In this case, EVs enhance post-burn immune dysfunction, and could thus develop into a novel therapeutic target in severe burn-injury treatment. It would be thus interesting to evaluate in future studies whether corneal burn injury alters EV release by the corneal cells or renders them more receptive to EV incorporation. Future studies are needed to fully dissect the mechanism of methylcellulose-embedded EV uptake and its action on corneal cells, using fluorescence/luminescence reporters [72]. We cannot presently rule out that corneal tissue damage may alter the release of endogenous EVs, or may alter the uptake of exogenous ones.

## 5. Conclusions

In the present study, we demonstrate, to our knowledge for the first time, how BMSC-EVs, embedded in methylcellulose, can modulate corneal repair dynamics in an alkali-induced corneal burn model, by generating an anti-inflammatory, pro-survival environment, which are key events in preventing new angiogenesis formation. No side effects of the non-autologous EVs were noted during the study period, as EVs were not internalized in the non-damaged corneas, suggesting that the BMSC-EVs do not penetrate the intact or repaired corneal epithelium. Thus, BMSC-EVs are promising as a new cell-free approach for intervening on burn wounds in the avascularized region of the eye and deserve further investigation. It would be important to address the combination of BMSC-EVs with different ophthalmic solutions or to functionalize EVs to potentiate their regenerative functions in re-establishing corneal homeostasis after burn injuries.

## Data Availability

Not applicable.

## Supplementary Materials

The following supporting information can be downloaded at: https://www.mdpi.com/article/10.3390/cells11233892/s1, Figure S1: Alkali-burn mouse model set-up and MRI assessment of corneal damage over time. Following this, 7T MRI T2w—images of corneal damage following alkali treatment at days 0, 1, 3, 7 and 14. Red arrow indicates the injured eye of animal models with application of NaOH paper disc for 30 s. Inset shows visible corneal damage at day 14 post alkali burn.; Figure S2: Confocal microscopy (Leica Sp5) images of corneal whole mounts, following treatment of the injured corneas with PKH26-labelled (Red) MSC-EVs at 40× magnification (scale bar: 100 nm). A zoomed insert is shown for detail (scale bar: 20 nm). F-actin and nuclei were stained with phalloidin-FITC (green) and DAPI (blue), respectively. The axes represent 3D corneal structure. Figure S3: MRI-based corneal angiogenesis evaluation with Gd-AAZTA-MADEC set-up: (A) Chemical structure of Gd-AAZTA-MADEC. (B) Representative region of interest (ROI) drawn on a 1T MRI image of cornea. Arrow indicates quantified region. (C) Representative MRI signal enhancement between injured and uninjured contralateral eyes followed for 4 h after injection of Gd-AAZTA-MADEC at day 3, where signal intensities post- Gd-AAZTA-MADEC injection were normalized against signal intensities measured in the same regions before the injection (time = 0). Table S1: qPCR primer sequences using SYBR Green^®^ technology for *Mus musculus* (Mm) and *Homo sapiens* (Hs).
