# Supplementary material for: Bone Marrow Mesenchymal Stromal/Stem Cell-Derived Extracellular Vesicles Promote Corneal Wound Repair by Regulating Inflammation and Angiogenesis
Source: Cells. 2022 Dec 2;11(23):3892. doi: 10.3390/cells11233892 (PMC9736484; doi:10.3390/cells11233892)
Supplement: Supplementary file 1 [file cells-11-03892-s001.zip › cells-2026190-supplementary.pdf]

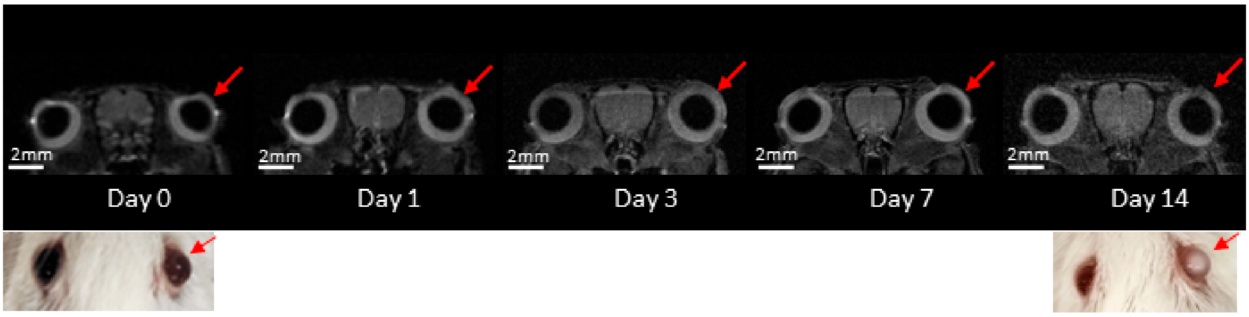

Figure S1. Alkali-burn mouse model set-up and MRI assessment of corneal damage over time. 7T MRI T_{2w} -images of corneal damage following alkali treatment at Days 0, 1, 3, 7 and 14. Red arrow indicates the injured eye of animal models with application of NaOH paper disc for 30 sec. Inset shows visible corneal damage at Day 14 post alkali-burn.

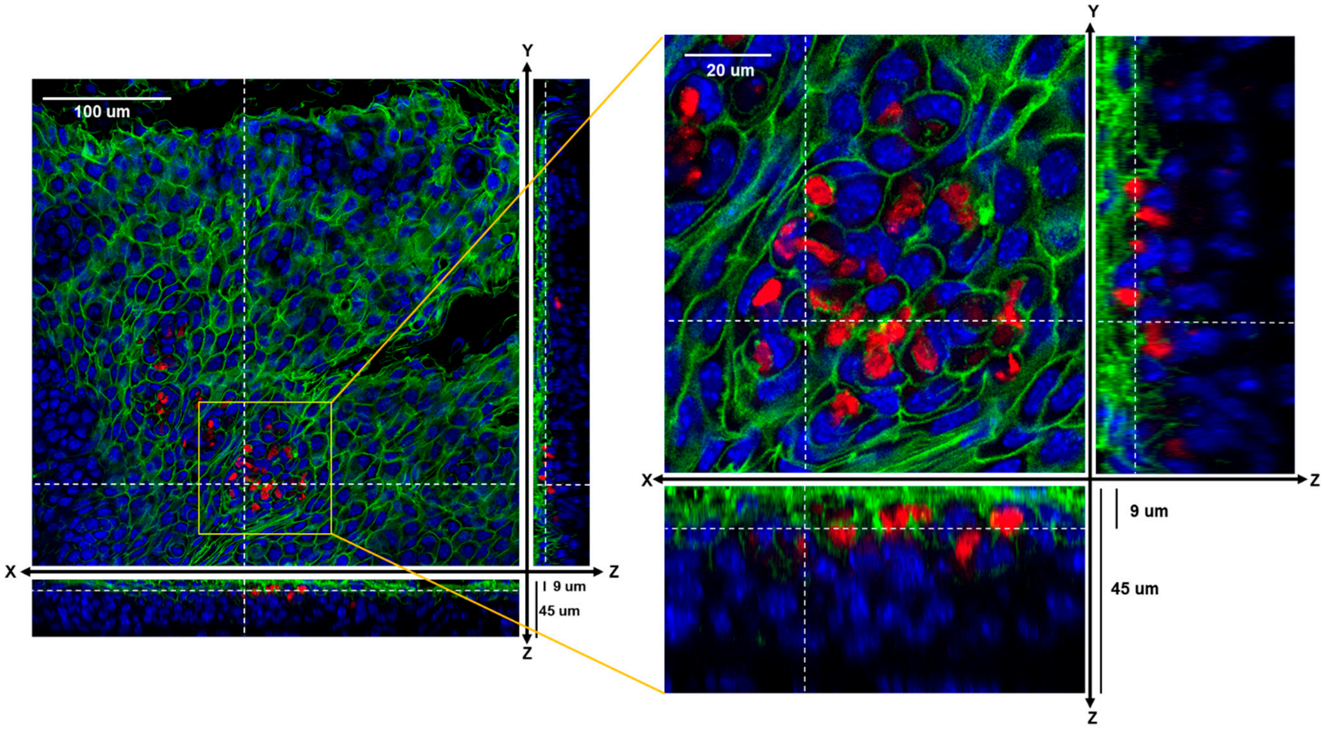

Figure S2: Confocal microscopy (Leica Sp5) images of corneal whole mounts, following treatment of the injured corneas with PKH26-labelled (Red) MSC-EVs at 40× magnification (scale bar: 100nm). A zoomed insert is shown for detail (scale bar: 20nm). F-actin and nuclei were stained with phalloidin-FITC (green) and DAPI (blue), respectively. The axes represent 3D corneal structure.

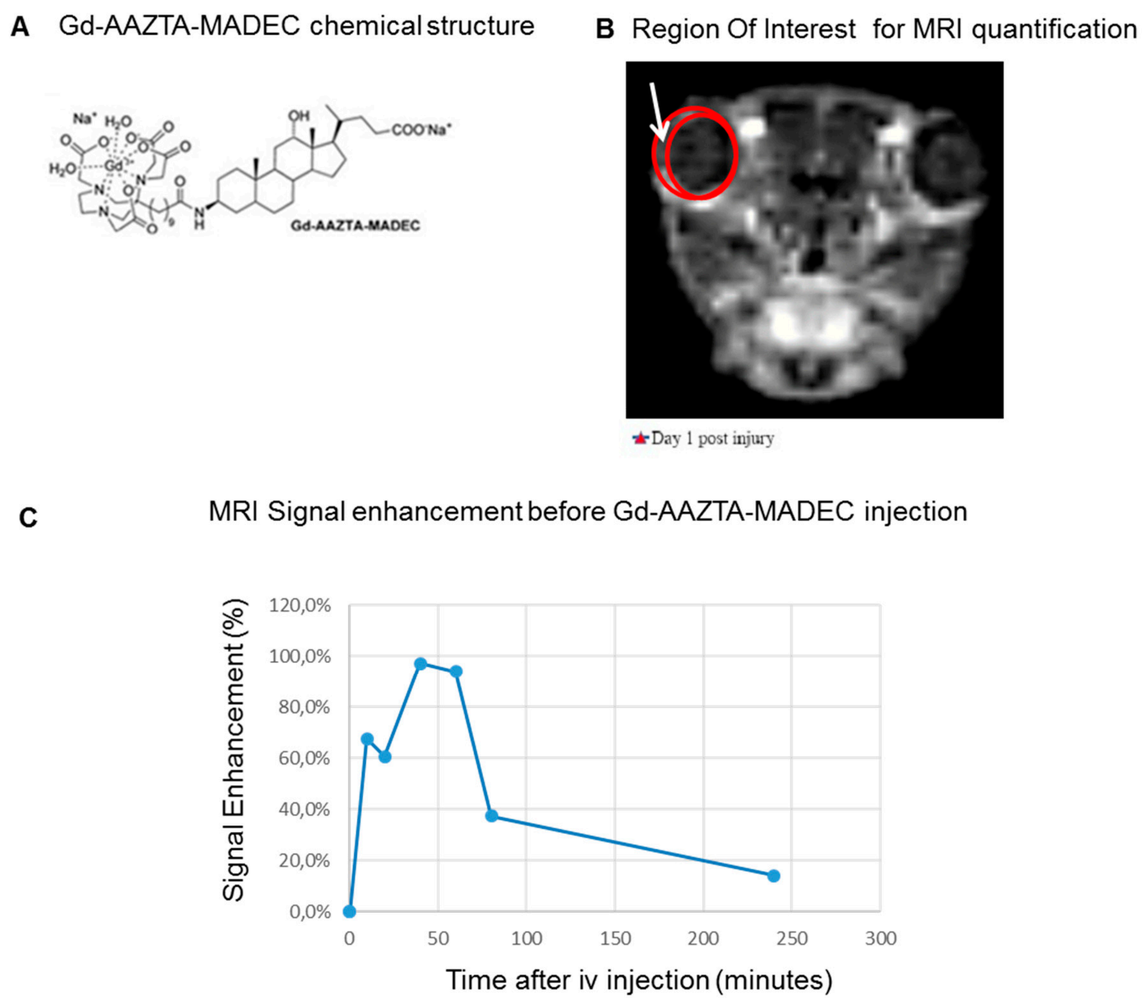

Figure S3: MRI-based corneal angiogenesis evaluation with Gd-AAZTA-MADEC set-up (A) Chemical structure of Gd-AAZTA-MADEC. (B) Representative Region of Interest (ROI) drawn on a 1T MRI image of cornea. Arrow indicates quantified region. (C) Representative MRI signal enhancement between injured and uninjured contralateral eyes followed for 4 h after injection of Gd-AAZTA-MADEC at Day 3, where Signal Intensities post-Gd-AAZTA-MADEC injection were normalized against signal intensities measured in the same regions before the injection (time=0).

Table S1: qPCR primer sequences using SYBR Green® technology for *Mus musculus* (*Mm*) and *Homo sapiens* (*Hs*).

*Mm*_PRIMER	Forward Sequence	Reverse Sequence
Inflammation gene		
Tnfa	ACTTCTGCTCTAGGGTGAGTGC	GGCCAGGGGGTATTCTTG
Il1b	AGTTGACGGACCCCAAAAG	AGCTGGATGCTCTCATCAGG
Il1r2	GCAAGAAGCAGCAAGGTACA	CCGCACCAACTTCCTGAG
Il6	GCTACCAAACTGGATATAATCAGGA	CCAGGTAGCTATGGTACTCCAGAA
Spp1 (Opn)	GGAAACCAGCCAAGGTAAGC	TGCCAATCTCATGGTCGTAG
Tgfβ	TGGAGCAACATGTGGAACTC	GTCAGCAGCCGGTTACCA
Angiogenic gene		
Vegfa	AAAAACGAAAGCGCAAGAAA	TTTCTCCGCTCTGAACAAGG
Vegfd	GCAACTTTCTATGACACTGAAACAC	TCTCTCTAGGGCTGCATTGG
Flt1	GGCCCGGGATATTTATAAGA	CCATCCATTTTAGGGGAAGT
Flt4	TGTCACCTTCGACTGGGATT	TGCTGGAGAGTTCTGTGTGG
Kdr(Flk1)	GGGGTATGGAGACGAGCTG	GCACAGATCTGACTAAATTGCTG
Housekeeping		
Gapdh	AGCCCAATCGCTCAG	GCCCAATACGACCAA

*Hs*_PRIMER	Forward Sequence	Reverse Sequence
Inflammation gene		
TNFA	AGAACCGGCAAGAAATCAAG	CTGGTAGGAGTCCTTCTTGTCC
PTGS2 (COX2)	GCTTTATGCTGAAGCCCTATGA	TCCAACTCTGCTAGACATTTCC
IL1B	TACCTGTCCTGCGTGTTGAA	TCTTTGGGTAATTTTTGGGATCT
Housekeeping		
GAPDH	AGCCACATCGCTCAGACAC	GCCCAATACGACCAAATCC
